# The effects of diurnal intermittent fasting on proinflammatory cytokine levels while controlling for sleep/wake pattern, meal composition and energy expenditure

**DOI:** 10.1371/journal.pone.0226034

**Published:** 2019-12-10

**Authors:** Aljohara S. Almeneessier, Abdulrahman A. BaHammam, Mohammed Alzoghaibi, Awad H. Olaish, Samar Z. Nashwan, Ahmed S. BaHammam

**Affiliations:** 1 Department of Family Medicine, College of Medicine, King Saud University, Riyadh, Saudi Arabia; 2 University Sleep Disorders Center, College of Medicine, King Sau University, Riyadh, Saudi Arabia; 3 King Abdulaziz & his companies Foundation for giftedness & creativity, Manarat Al Riyadh School, Ministry of Education, Riyadh, Saudi Arabia; 4 Department of Physiology, College of Medicine, King Saud University, Riyadh, Saudi Arabia; 5 The Strategic Technologies Program of the National Plan for Sciences and Technology and Innovation, Saudi Arabia (MED511-02-08), Riyadh, Saudi Arabia; University of Lübeck, GERMANY

## Abstract

**Purpose:**

This study aimed to assess the effect of diurnal intermittent fasting (DIF) during and outside of the month of Ramadan on plasma levels of interleukin (IL)-1β, IL-6, and IL-8, while controlling for sleep/wake pattern, sleep length and quality, meal composition, energy consumption and expenditure, and light exposure. DIF outside of the month of Ramadan was performed to evaluate the effect of DIF in the absence of the way of life accompanying Ramadan.

**Methods:**

Twelve healthy male volunteers with a mean age of 25.1 ± 2.5 years arrived to the sleep laboratory on 4 times: 1) adaptation, 5 weeks before *Ramadan*; 2) 4 weeks before *Ramadan* while performing DIF for 1 week (fasting outside of Ramadan; FOR); 3) 1 week before *Ramadan* (non-fasting baseline; non-fasting BL); and 4) After completing 2 weeks of *Ramadan* while performing DIF. Plasma levels of cytokines were assessed using enzyme-linked immunoassays at 22:00, 02:00, 04:00, 06:00, and 11:00.

**Results:**

During DIF, there was a significant decrease in the levels of cytokines, particularly, IL-1β and IL-6, in most measurements compared to non-fasting BL. This reduction was more obvious during the FOR period. There were no significant changes in the circadian phase of the measured cytokines reflected by the acrophase of the measured variables during fasting (FOR and Ramadan) compared to non-fasting BL.

**Conclusion:**

Under controlled conditions, DIF led to significantly decreased plasma levels of cytokines (IL-1β, IL-6, and IL-8), particularly IL-1β and IL-6 across 24 h. DIF had no effect on the circadian patterns of the measured cytokines as shown by cosinor analysis.

## Introduction

Recently, great interest had been developed on the effect of intermittent fasting on cadriometabolic risk, particularly, diurnal intermittent fasting (DIF) [1]. DIF is practiced by hundreds of millions of Muslims during the month of Ramadan [2]. The effects of DIF on human health have attracted the attention of investigators in the past few years. DIF is not similar to caloric restriction (CR), in which caloric intake is reduced for long periods, but meal frequency is preserved [2]. During Ramadan DIF, fast performers abstain from food, drinks, and smoking from dawn to sunset for a period of one month. DIF during Ramadan fasting can be looked at as a time-restricted feeding (between dawn and sunset) protocol with no calorie restriction. Traditionally, 2 main meals are taken at night during Ramadan; the first after sunset and the second before dawn, where performers usually obtain adequate sleep at night before the pre-dawn meal [3]. This practice has a religious dimension that aims to motivate performers to rise early, before dawn, for the pre-dawn meal and dawn prayer. However, recent studies have shown that Ramadan month is associated with several lifestyle changes that might influence the health of fast performers, such as changes in sleep/wake schedule, reduced nocturnal sleep duration, reduced physical activity, increased caloric intake, and changes in meal contents [2]. Therefore, data from studies assessing the health effects of CR or other types of fasting cannot be extrapolated to the Ramadan DIF [1].

Cytokines are small glycoproteins produced by several cell types, especially white blood cells, which regulate immunity and response to inflammation. Their persistent elevation has negative effects on various organs and bodily functions, such as increasing the risk of diabetes, metabolic syndrome, and cardiovascular diseases [4]. Studies in animal models have shown that low-calorie intake reduces the levels of proinflammatory cytokines [5]. A few studies have examined inflammatory biomarkers during Ramadan DIF. A recent meta-analysis of 10 studies conducted on DIF performers during Ramadan demonstrated a small reduction in inflammatory biomarkers during fasting [6].

However, previous studies collected only a single morning blood sample for evaluation of proinflammatory cytokine levels. Additionally, previous studies that measured proinflammatory cytokines during DIF were performed without controlling for lifestyle changes that might affect the measured biomarkers, such as caloric intake [7], sleep duration [8], changes in the biologic clock [9], circadian variation of the levels of biomarkers [10], and energy expenditure [11]. Therefore, the obtained results might be influenced by these factors and not reflect the pure effect of DIF. In order to assess the effects of DIF during Ramadan on proinflammatory cytokine levels, an experimental design that has good control of the associated lifestyle changes is mandatory.

To account for the variation in the levels of the biomarkers with the biological clock, plasma levels of cytokines were measured at 22:00, 02:00, 04:00, 06:00, and 11:00 [10, 12, 13]. Interleukin (IL)-1β, IL-6, and Il-8 are major components of low-grade systemic inflammation and are important proinflammatory cytokines that predispose the development of cardiometabolic diseases [14]. IL-1β is a central mediator of inflammatory responses, involved in a range of cellular activities, including cell proliferation, differentiation, and apoptosis [15]. IL-6 is secreted by T-lymphocytes and macrophages and has a role in chronic inflammation and autoimmunity [16]. IL-8 is a proinflammatory cytokine that induces chemotaxis in target cells, mainly neutrophils and other granulocytes [17, 18].

Based on the above, we hypothesized that DIF reduces proinflammatory cytokine levels. Therefore, this study was designed to assess the effect of DIF during and outside of the month of Ramadan on plasma levels of IL-1β, IL-6, and IL-8 while controlling for the sleep/wake pattern, sleep length and quality, meal composition, energy consumption, and light exposure. DIF outside of the month of Ramadan was performed to evaluate the effect of DIF on plasma levels of cytokines in the absence of the lifestyle changes accompanying Ramadan. This study allowed the investigaotrs to assess the effects of DIF on proinflammatory cytokine levels.

## Methods

### Study subjects

The research project was approved by the institutional review board of the College of Medicine at King Saud University and written informed consent was obtained from all participants. Twelve non-smoking, healthy volunteer males between the ages of 20 and 30 years were included. Participants were recruited through printed and electronic advertisements on notice boards at the University campus. The advertisement indicated the research objectives, protocol, and duration. Exclusion criteria included taking any medications (prescription or over-the-counter), drinking alcohol, being on diet, body mass index (BMI) ≥ 30 kg/m^2^, and shift workers. All participants were interviewed, and their electronic medical files were reviewed to rule out medical, sleep, or psychiatric disorders. Before final recruitment, the sleep/wake schedule of the participants was monitored using wrist actigraphy for one week to confirm a regular sleep pattern and morning chronotype. A regular sleep/wake schedule was considered if the daily variability in bedtime and rise time were less than 1 hour [19].The work schedules of the participants during weekdays were from 07:30 to 16:30 outside of *Ramadan* and from 10:00 to 15:00 during *Ramadan*.

### Study protocol

**Fig 1:**
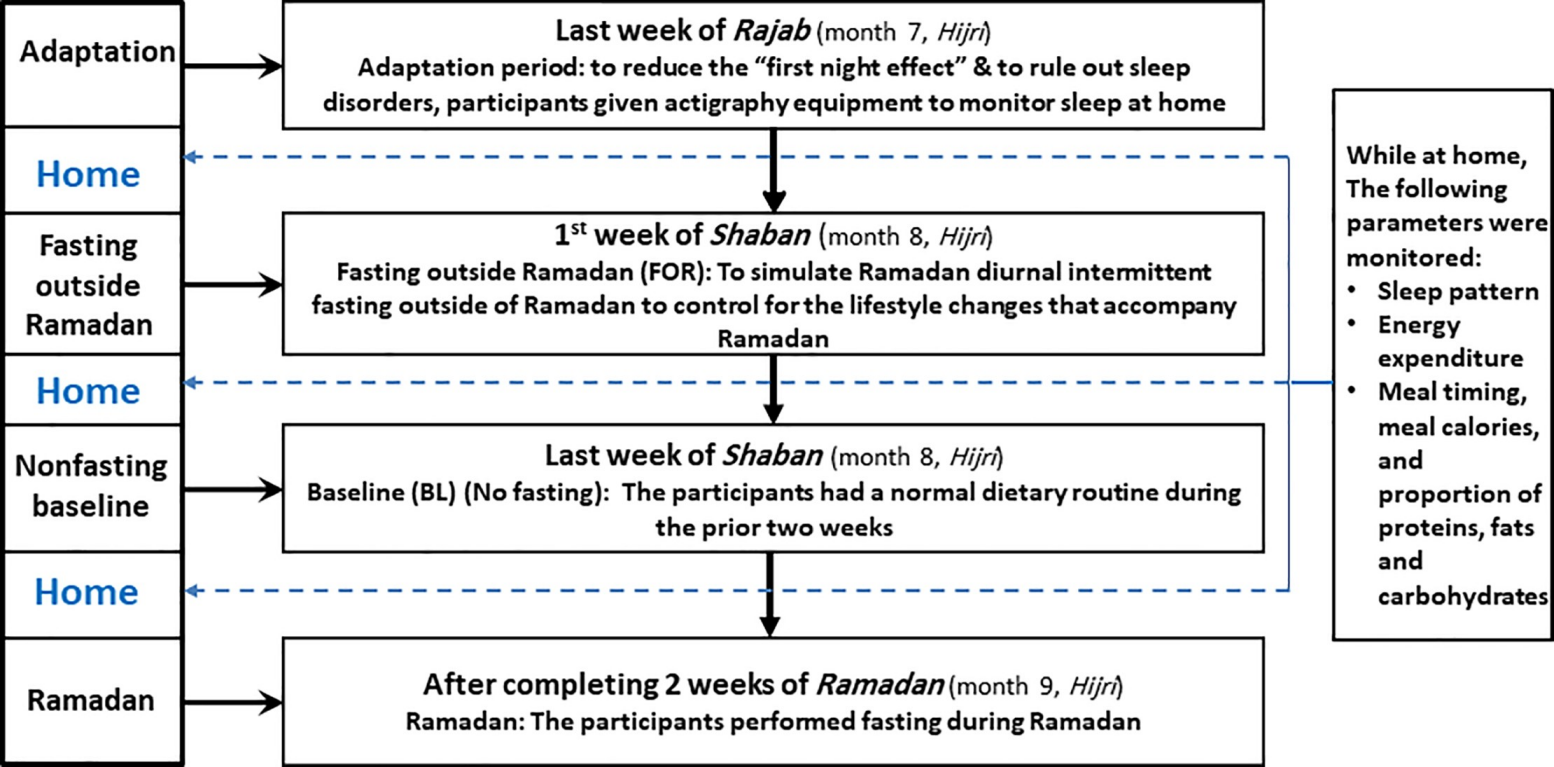
Study protocol.

We have used and described the applied protocol in detail in previous studies [19, 20]. Fig 1 illustrates the protocol of the study. The study was conducted following the *Hijri* (Islamic) calendar, which follows the lunar system in which the year is 11 days shorter than the Gregorian year [21]. The study was conducted during the last week of month number 7 (*Rajab*), month number 8 (*Shaban*), and the first 2 weeks of month number 9 (*Ramadan*) of the *Hijri* year 1439, which in the Gregorian calendar corresponds to the period from April 9 to May 29, 2018.

As per the study protocol, the volunteers reported to the laboratory at 18:00 and spent approximately 24 h in the laboratory on 4 occasions as detailed below. During each stay, an overnight polysomnography (PSG) was performed to measure sleep parameters:

1. **The last week of month number 7 (*Rajab*)**: The aim of this visit was to ensure adaptation to the environment and equipment used in order to minimize the “first night effect” [22].
2. **The first week of month number 8 (*Shaban*)**: Participants were asked to perform diurnal fasting (i.e., abstain from food and drink on the first week of Shaban from dawn to sunset) for one week and were admitted to the laboratory for monitoring on the last day of the week. This fasting protocol was labeled as “*Fasting outside Ramadan*” (FOR) and aimed to assess the effects of DIF on the measured bio-inflammatory markers outside of *Ramadan* to eliminate the effect of lifestyle changes that accompany *Ramadan* [23]. This fasting week was ended 3 weeks before *Ramadan* to ensure an adequate wash-out period and to avoid any carryover effects.
3. **The last week of month number 8 (*Shaban*)**: During the last 3 weeks of the month of *Shaban*, participants were not fasting. This period was termed “*non-fasting baseline* (BL).” Participants were admitted to the laboratory for approximately 24 h for monitoring between days 21 and 28 of the month.
4. **After completing 2 weeks of *Ramadan* (month 9, Hijri)**: Participants performed diurnal fasting (from dawn to sunset) during the first 2 weeks of *Ramadan*. This period was called “*Ramadan fasting*.” Participants were admitted to the laboratory for approximately 24 h for monitoring between days 14 and 17 of the month.

### Monitoring at home

During the study period, participants were instructed to follow the same sleep/wake pattern and physical activity. The sleep/wake pattern at home was objectively monitored using actigraphy. Additionally, energy expenditure was monitored via the SenseWear Pro Armband^™^ (Body Media, Pittsburgh, PA, USA) (see below for details). Meals at home were specified to match the meals in the laboratory for the fasting (FOR and Ramadan) and non-fasting (BL) periods concerning mealtime and meal composition (proportion of proteins, carbohydrates, and fats) and calories, and a nutritionist worked with the participants to achieve that goal. A daily checklist was completed by the participants to ensure abiding by the set protocol.

### Protocol in the laboratory

**• Fasting and meal-timings protocol**

During their stay in the laboratory, participants performed fasting from dawn to sunset. During non-fasting BL, participants received 3 meals; dinner at 20:30, breakfast at 07:15, and lunch at 12:00 (mid-day). During FOR and *Ramadan*, a light meal was served at sunset to break the fast, followed by a dinner meal at 21:00 and a pre-dawn (*Suhur*) meal 30 min before dawn time. During the study period, sunset and dawn times ranged from 18:23–18:35 and 04:02–03:35, respectively. A fixed caloric intake (35 Kcal/kg/24 h) and a fixed proportion of proteins, fats, and carbohydrates were served.

**• Light intensity in the laboratory**

From 18:00 until bedtime, the light level was maintained at 50 lux. During the pre-dawn meal, the light was kept < 30 lux [24]. From bedtime until rising time (PSG recording), all lights were switched off and light intensity was maintained at < 1 lux. Light intensity was determined using a Spectral Star Light Meter LX-1 (Japan).

**• Energy expenditure in the laboratory**

Energy expenditure in the laboratory was monitored using the SenseWear Pro Armband^™^ (Body Media, Pittsburgh, PA, USA). The armband is a portable device worn around the right arm.

The device has sensors that continuously measure skin temperature, galvanic skin response, heat flux, and movements and uses a validated advanced algorithm to estimate total energy expenditure [25, 26].

**• Sleep monitoring in the laboratory**

Participants were instructed to avoid napping during the day of admission to the laboratory. During non-fasting BL, bedtime was at 23:00 and rise time at 07:00. During FOR and *Ramadan*, bedtime was at 23:00. During *Ramadan*, participants were awakened at 03:05 for the pre-dawn meal, and participants resumed sleep from 03:50 until 07:45. During FOR, participants were awakened at 03:45 for the pre-dawn meal (to account for the shift in dawn time), and participants resumed sleep from 04:30 until 07:45.

Sleep monitoring was performed using Alice 6 diagnostic equipment (Philips/Respironics, Inc., Murrysville, PA, USA). The following parameters were reported in the results: sleep efficiency (the percentage of time spent asleep while in bed); arousal index, which is a measure of sleep disruption reporting on the number of arousals per hour of sleep; and “stage shifts,” which reports on the number of changes in sleep stages from lights out to lights on. Scoring was performed according to the American Academy of Sleep Medicine scoring criteria [27].

### Blood samples collection and measurement of cytokine levels

At 20:00, a cannula was placed in a vein in the antecubital fossa of the arm, in order to draw blood without disturbing sleep. Five blood samples were collected from each participant when in the laboratory in the 3 periods: FOR, non-fasting BL, and *Ramadan*. Blood samples for IL-1β, IL-6, and IL-8 were collected at 22:00, 02:00, 04:00, 06:00, and 11:00 h. After blood samples were collected, they were immediately centrifuged at 4°C and then stored at -70°C.

Levels of interleukins were determined using enzyme-linked immunoassays (DIAsource ImmunoAssays S.A., Ottignies-Louvain-la-Neuve, Belgium). The assay sensitivity for IL-1β was 0.35 pg/mL. The intra-assay and inter-assay coefficients of variation were <2.4% and <5%, respectively. The assay sensitivity for IL-6 was 2 pg/mL. The intra-assay and inter-assay coefficients of variation were <4.3% and <5.4%, respectively. The assay sensitivity for IL-8 was 1.1 pg/mL. The intra-assay and inter-assay coefficients of variation were <3.6% and <13.1%, respectively. All measurement procedures were performed according to the manufacturer’s instructions.

Serum glucose level was measured for all participants at 15:30 when in the laboratory.

### Statistical analysis

Continuous variables are presented in the text, tables, and figures as the mean ± standard deviation. Metabolic equivalents (METs) were used to express energy expenditure during day and night. Average METs were calculated to obtain an overall hourly average for each day.

Cosinor analysis for all measured cytokines was performed using a 24-h cosinor rhythmometry model

$$f(t)=M+A*Cos(\frac{2\pi t}{T}+\emptyset ),$$

where M = mesor; A = amplitude; Ø = acrophase, and T = 24 h, to obtain the best estimates of the acrophase for IL-1β, IL-6, and IL-8 [28]. The model was executed for the data of each participant during the 3 periods. Comparisons of the non-fasting BL, FOR, and *Ramadan* fasting groups were performed using repeated measure analysis of variance (ANOVA). Repeated measures ANOVA on ranks test was used if the normality test failed. Post hoc analysis (Holm-Sidak) was used to estimate the significance of differences between individual groups if normality test was achieved and psot hoc analysis (Tukey) was sued with the One-way repeated measures ANOVA on ranks. Data of cytokines’ levels at each measured time were presenter as Box & Whisker Plot. This plot allows the presentation of lower extreme, lower quartile, median, upper quartile, and upper extreme. The upper part of the box represents the 75^th^ percentile, the horizontal line in the box represents the median, and the lower part of the box represents the 25^th^ percentile. The whiskers (lines) of the box represents the smallest and largest values that are not minor or extreme outlying values.

A p-value of < 0.05 was considered significant. SigmaStat version 3.5 software (Dundas Software LTD., wpcubed GmbH, Germany) was used for the analyses.

## Results

The mean age of participants was 25.1 ± 2.5 years and the mean BMI was 23.4 ± 3.5 kg/m^2^. Body weight remained stable during the 3 study periods (69.3 ± 6.6 kg, 69.6 ± 5.7 kg, and 69.1 ± 6.8 kg for non-fasting BL, FOR, and *Ramadan*, respectively, *p* = 0.9). In addition, no significant variations were detected in serum glucose levels while in the laboratory, in the 3 study periods (5.8 ± 0.5, 5.6 ± 0.5, and 5.5 ± 0.6 mmol/L during non-fasting BL, FOR, and *Ramadan*, respectively, *p* = 0.4).

Table 1 displays the sleep pattern and energy expenditure at home. There was a significant delay in bedtime and rise time during *Ramadan* compared to the non-fasting BL and FOR periods. However, there were no significant changes observed in sleep duration between the 3 periods. Additionally, there was a significant reduction in energy expenditure during *Ramadan* when compared to the BL period. However, no significant changes in energy expenditure were noted during FOR. All participants followed the meal timing and composition protocol during FOR; however, only two of them followed the food protocol during *Ramadan*.

**Table 1:** Sleep/wake pattern while at home during the study periods, measured via actigraphy.

Measured variables	Non-fasting BL	FOR	*Ramadan*	p-value
Mean Bedtime	00:39	01:05	02:33*	0.003
Mean Wake-up time ± SD (in h)	05:35	05:50	08:56*	0.031
*n*TST (h)	5.9±1.3	5.7±1.4	6.3±1.6	0.6
*n*TST + NAP (h)	6.8±1.1	6.7±1.9	7.2±2.5	0.8
METs	1.89±0.18	1.77±0.19	1.71±0.15*	0.02

*n*TST: nocturnal total sleep time; NAP: daytime naps

*Statistically significant difference between non-fasting BL and *Ramadan*. No difference observed between non-fasting BL and FOR.

Table 2 presents the measured sleep parameters and energy expenditure while in the laboratory. No differences were observed in sleep parameters reflecting sleep quality while in the laboratory between the 3 study periods, including, total sleep time, sleep efficiency, arousal index, stage shifts, and sleep latency. Sleep efficiency was slightly lower than normal during all periods which might reflect the effect of drawing blood samples with the intravenous cannula in the antecubital fossa 5 times during sleep.

**Table 2:** Sleep parameters and energy expenditure while in the laboratory during the 3 study periods.

Measured variables	Non-fasting BL	Fasting outside Ramadan	Ramadan	p-value
**Sleep parameters during monitoring at the laboratory using polysomnography**				
Total sleep time (min)	359.5 ± 41.9	351.5 ± 49.9	341.7 ± 52.3	0.7
Sleep efficiency (%)	79.3 ± 9.1	78.1 ± 8.4	75.8 ± 10.2	0. 6
Sleep latency (min)	31.3 ± 18.9	28.5 ± 16.7	30.7 ± 22.4	0.9
Arousal index (arousal/hr)	6.7 ± 3.5	7.1 ± 4.7	8.2 ± 3.9	0.6
Stage shifts	69.1 ± 19.7	72.1 ± 18.3	73.4 ± 20.7	0.9
**Energy expenditure based on the SenseWear Pro Armband**				
METs	1.5 ± 0.1	1.4 ± 0.2	1.4 ± 0.1	0.2
METs (Day)	1.5 ± 0.1	1.4 ± 0.2	1.4 ± 0.2	0.3
METs (Night)	1.4 ± 0.1	1.4 ± 0.2	1.4 ± 0.1	1

METs: metabolic equivalents; stage shifts: total number of changes in sleep stage from the sleep-onset to rise time.

No changes were noted in energy expenditure (expressed by METs) between day and night during the 3 monitoring periods in the laboratory.

**Fig 2:**
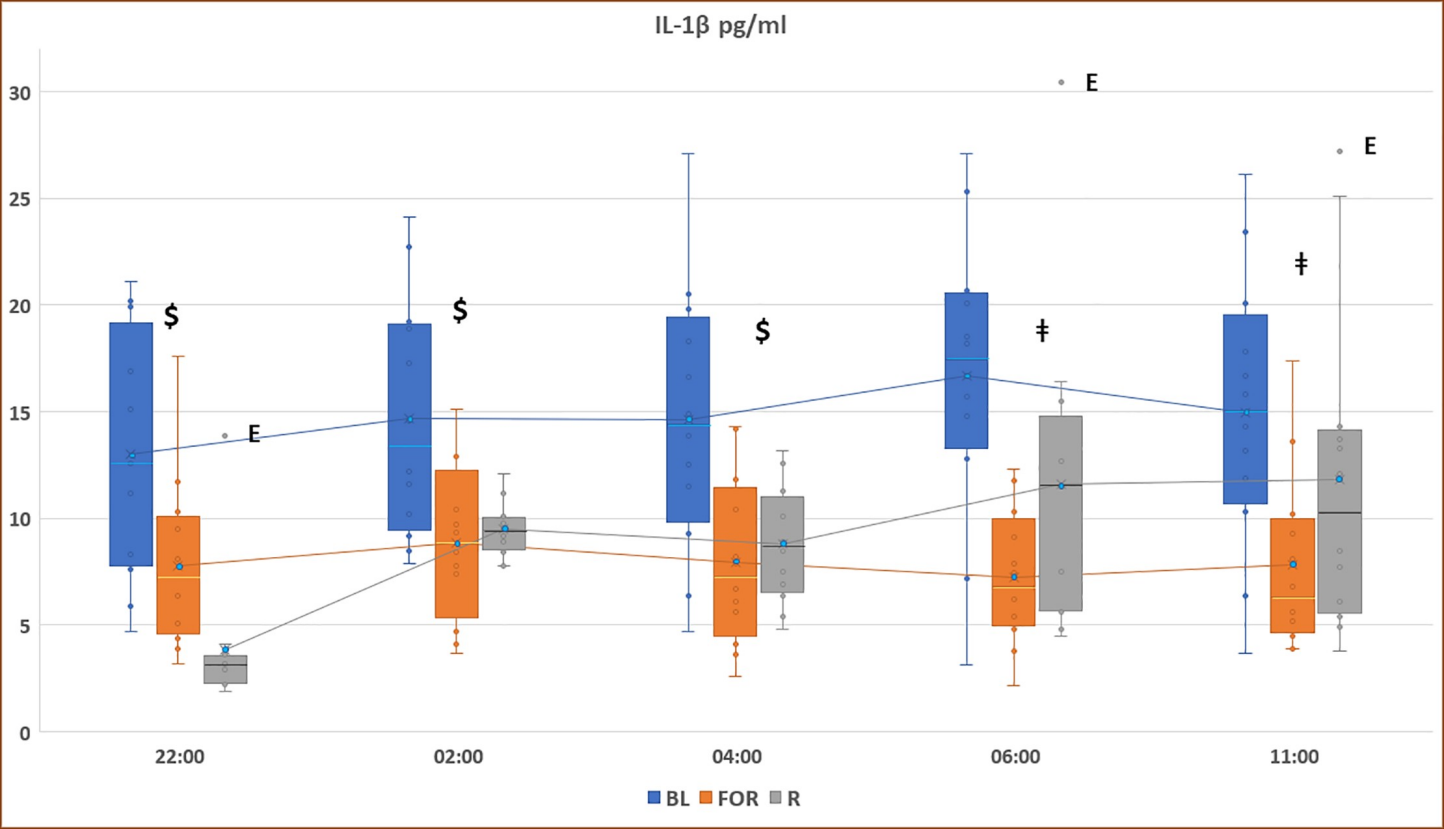
Circadian pattern of plasma IL-1β concentrations at non-fasting baseline (non-fasting BL), fasting outside *Ramadan* (FOR), and *Ramadan*. Plasma levels were presented as box and whisker plot. The upper part of the box represents the 75^th^ percentile, the horizontal line in the box represents the median, the lower part of the box represents the 25^th^ percentile, and the blue circle represents the mean. The whiskers (lines) of the box represents the smallest and largest values that are not minor or extreme outlying values. $ indicates significance between both Ramadan and fasting outside of Ramadan and baseline measurements (BL), (p < 0.05). ǂ indicates significance between FOR and BL, (p < 0.05). E: extreme outlier.

Fig 2 displays the pattern of IL-1β concentrations during non-fasting BL, FOR, and *Ramadan*. There was a significant decrease in IL-1β levels during FOR compared to the non-fasting BL period at 06:00, 11:00, 22:00, 02:00, and 04:00 (p<0.05). Additionally, there was a significant reduction in IL-1β levels during Ramadan compared to the non-fasting BL period at 22:00, 02:00 and 04:00 (p<0.05).

**Fig 3:**
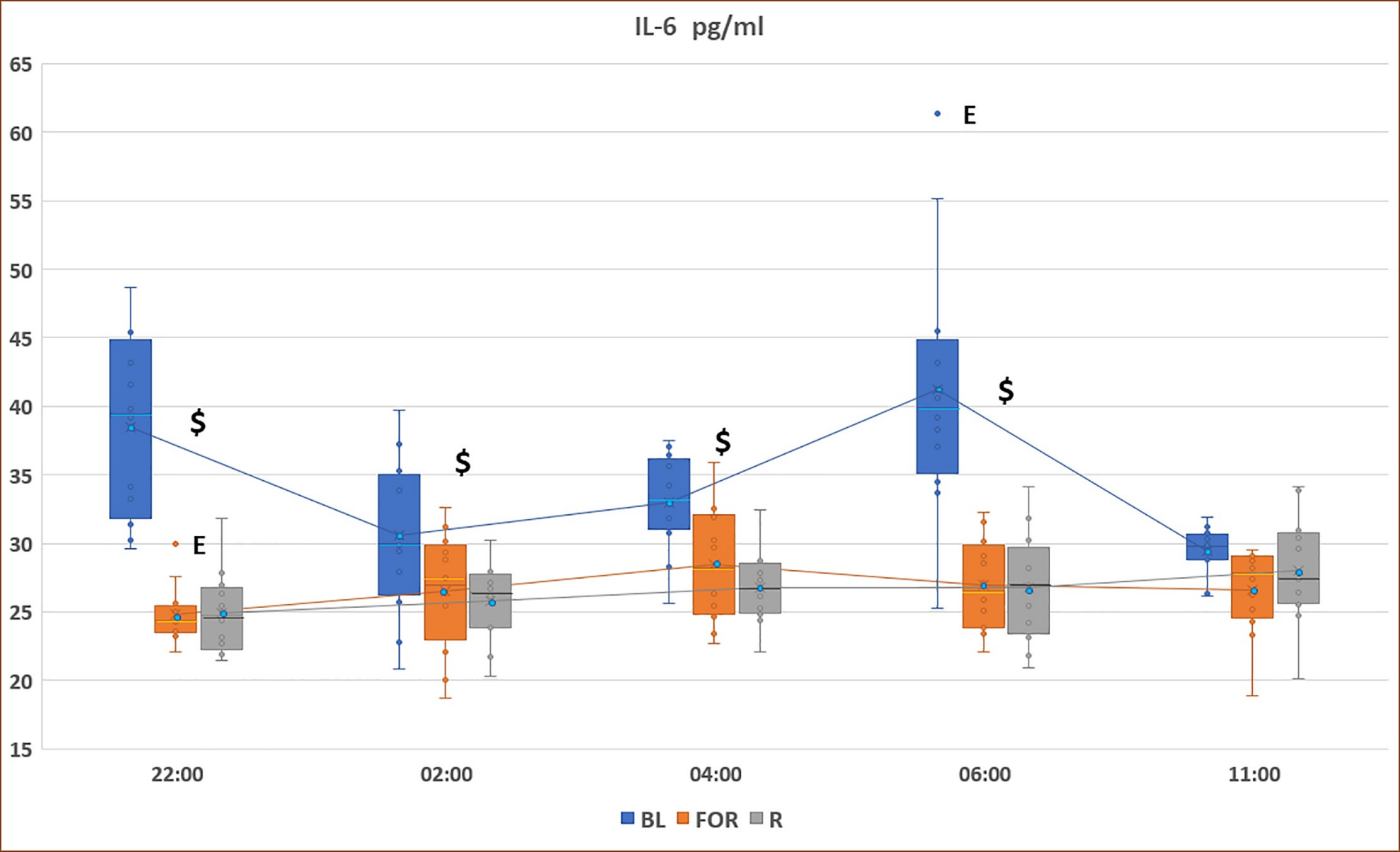
Circadian pattern of plasma IL-6 concentrations at non-fasting BL, FOR, and *Ramadan*. Plasma levels were presented as box and whisker plot. The upper part of the box represents the 75^th^ percentile, the horizontal line in the box represents the median, the lower part of the box represents the 25^th^ percentile, and the blue circle represents the mean. The whiskers (lines) of the box represents the smallest and largest values that are not minor or extreme outlying values. $ indicates significance between both Ramadan and fasting outside of Ramadan and baseline measurements (BL), (p < 0.05). ǂ indicates significance between FOR and BL, (p < 0.05). E: extreme outlier.

Fig 3 demonstrates the pattern of IL-6 concentrations during non-fasting BL, FOR, and *Ramadan*. There was a significant reduction in IL-6 levels during FOR and Ramadan compared to the non-fasting BL period at 06:00, 22:00, 02:00, and 04:00 (p<0.05).

**Fig 4:**
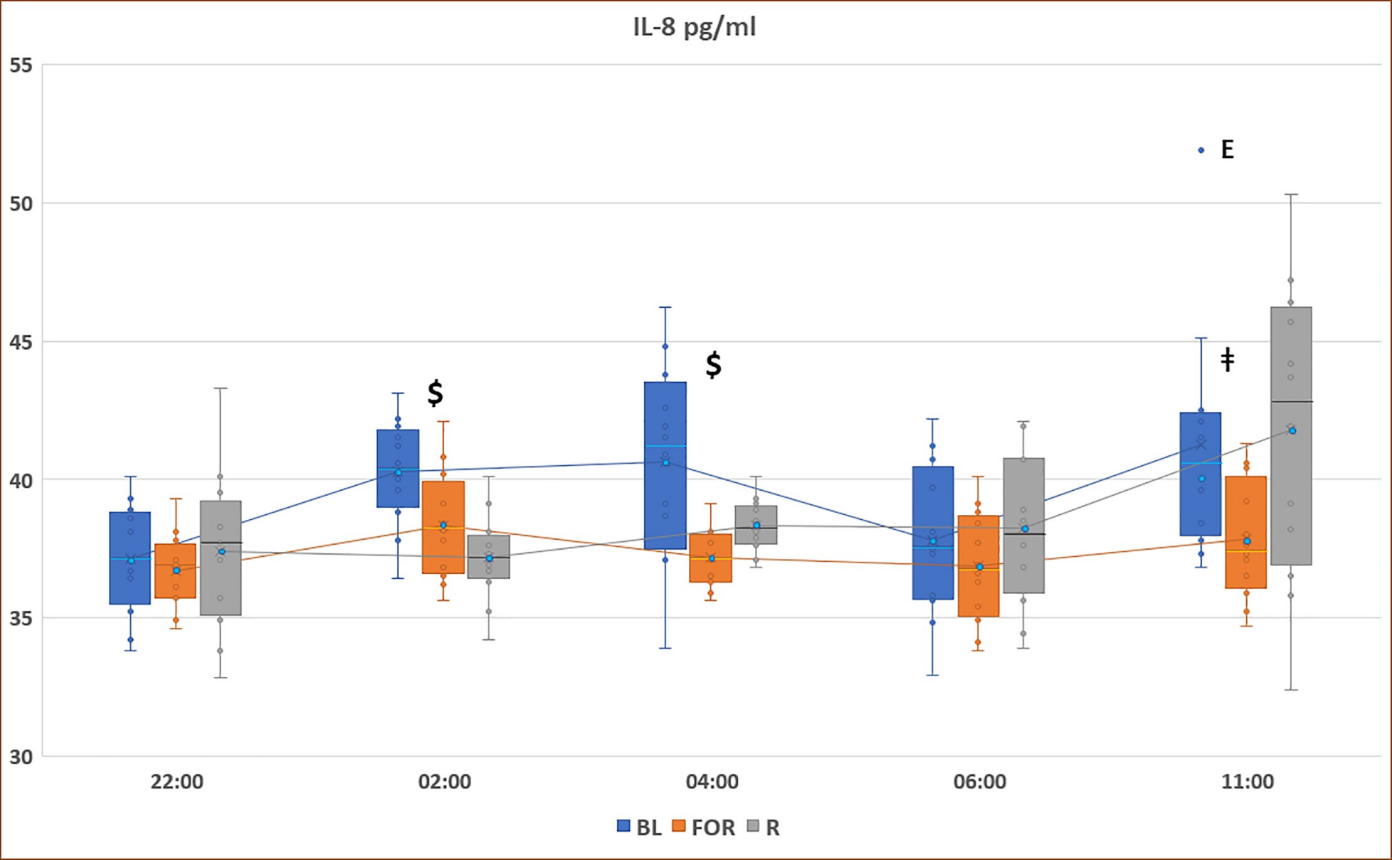
Circadian pattern of plasma IL-8 concentrations at non-fasting BL), FOR, and Ramadan. Plasma levels were presented as box and whisker plot. The upper part of the box represents the 75^th^ percentile, the horizontal line in the box represents the median, the lower part of the box represents the 25^th^ percentile, and the blue circle represents the mean. The whiskers (lines) of the box represents the smallest and largest values that are not minor or extreme outlying values. $ indicates significance between both Ramadan and fasting outside of Ramadan and baseline measurements (BL), (p < 0.05). ǂ indicates significance between FOR and BL, (p < 0.05). E: extreme outlier.

Fig 4 presents the pattern of IL-8 concentrations during non-fasting BL, FOR, and *Ramadan*. There was a significant reduction in IL-8 levels during FOR compared to the non-fasting BL period at 11:00, 02:00, and 04:00 (p<0.05). With regard to Ramadan, there was a significant reduction in IL-8 levels during Ramadan compared to the non-fasting BL period at 02:00 and 04:00 (p<0.05).

Table 3 shows the cosinor analysis of IL-1β, IL-6, and IL-8 plasma levels. There were no significant changes in the circadian phase of the cytokines profile as reflected by acrophase. However, the amplitude of IL-6 during Ramadan was significantly lower than the value at BL (p = 0.038).

**Table 3:** Cosinor analysis summary of IL-1β, IL-6, and IL-8 circadian rhythms during baseline, baseline fasting, and Ramadan.

	*BL*	*FOR*	*Ramadan*	*p-value*
**IL-1β Circadian Rhythm**				
Amplitude	6.7 ± 5.3	8.3 ± 7	6.9 ± 2.9	0.941
Acrophase	4.1 ± 2.8	4 ± 2.5	4.1 ± 0.7	1.000
**IL-6 Circadian Rhythm**				
Amplitude	12 ± 7	4.4 ± 4.4	2 ± 0.6	0.038*
Acrophase	1.7 ± 1.1	3.8 ± 2.9	3.9 ± 0.5	0.203
**IL-8 Circadian Rhythm**				
Amplitude	2.8 ± 1.7	2.3 ± 1.9	4.1 ± 0.5	0.242
Acrophase	4.8 ± 1.9	3.1 ± 3.3	3.6 ± 0.3	0.563

Tukey Post Hoc multiple comparison

* BL vs. Ramadan (p = 0.038)

BL: Baseline data; FOR: fasting outside of Ramadan

## Discussion

This is the first study to assess the effects of DIF on the levels of selected proinflammatory cytokines during and outside of *Ramadan* using several readings at different times of day and night (24 h) to control for circadian rhythm effect. Additionally, this is the first study to objectively monitor both sleep and energy expenditure while at home as both factors may influence the levels of measured inflammatory cytokines. No changes were observed during FOR compared to BL in sleep and energy expenditure, which make the results during FOR more reflective of the true effect of DIF. However, during Ramadan, it is difficult to control for sleep pattern, physical activity, and meals while at home as working time and hours change and get shorter, and the whole system gets delayed in the whole society [29]. Additionally, during *Ramadan*, fasting performers stay awake until late at night to perform *Ramadan* night prayers [21]. The findings of the current study supported our hypothesis that DIF results in reduced levels of proinflammatory cytokines. Our results demonstrated a significant reduction of the levels of cytokines across 24 h during fasting compared to baseline, and particularly those of IL-1β and IL-6 during fasting outside of *Ramadan*. The greater reduction in cytokine levels during FOR compared to *Ramadan* suggested that DIF, per se, can lead to reduced proinflammatory cytokines in the absence of *Ramadan*-associated lifestyle changes.

Previous studies that assessed the effect of DIF on levels of proinflammatory cytokines during *Ramadan* reported only on the evaluation of a single morning sample of the measured biomarkers, not accounting for the diurnal rhythmicity of cytokine production [12, 30]. Moreover, currently available data have suggested that mealtime and meal composition might affect the levels of the measured inflammatory markers [31, 32]. For example, it has been shown in mice that postprandial IL-1β levels increase in response to a meal [33]. Similar findings have been demonstrated in humans, where an increase in plasma IL-1β levels after intake of a high fat content meal was reported [34]. Therefore, collecting a single sample in the early morning after a heavy pre-dawn meal may influence the levels of the measured markers. Additionally, shifting mealtimes to the night during DIF might have an effect on the biologic clock of the body and its physiological responses [1], and hence may affect the rhythmicity of the inflammatory biomarkers. To overcome this, taking several blood samples around the clock would be the most efficient way to assess the effects of DIF on the levels of serum cytokines. Additionally, all previous studies did not control for factors shown to affect the levels of the measured biomarkers, such as caloric intake [7], sleep duration [8], and energy expenditure [11].

Studies that assessed circadian rhythm and biological clock markers during Ramadan DIF without controlling for the above mentioned lifestyle changes that accompany Ramadan, demonstrated a shift delay in the body circadian rhythm [2]. Recent well-designed studies revealed that the body internal circadian clock influences the levels of cytokines [12]. Moreover, cytokines in human blood have been shown to display circadian rhythms. Therefore, diurnal rhythmicity of cytokine secretion has important implications for the timing of blood samples collection. Previous studies that assessed cytokine levels during DIF did not account for possible changes in the biologic clock of volunteers and did not assess the effects of DIF on the circadian pattern of cytokines. In the present study, we controlled for light exposure, as well as bedtime and rise time to avoid changes in the circadian clock of participants. Using the same protocol, we have previously demonstrated that there is no change in the circadian rhythm of melatonin [35]. Additionally, we used a cosinor model to assess the effect of DIF on the circadian pattern of the measured cytokines. The present study demonstrated no significant changes in the circadian phase (acrophase) of the measured cytokines during DIF. However, the amplitude of IL-6 was significantly lower during Ramadan compared to BL, which reflects a more reduction the circadian oscillation of IL-6 after 2 weeks of fasting compared to the reduction after 1 week of fasting (FOR period). This reduction in the amplitude of IL-6 rhythmicity may suggest that, at least in part, longer fasting might have a sustained inhibitory effect on inflammation [36]. Further studies are needed to support this hypothesis.

Weight loss has been proposed as a mechanism for the reduction of proinflammatory cytokine levels during DIF [37]. Therefore, it is important to monitor weight when assessing the impact of DIF on proinflammatory cytokine levels, as weight changes can be a major confounder affecting cytokine levels [38, 39]. In the present study, we enlisted volunteers with normal BMI and monitored their weight during the study period. Hence, weight loss could not be the sole mechanism explaining the reduction of cytokine levels. Additionally, both animal and human studies have suggested that CR results in a decrease in proinflammatory cytokine levels [5, 40]. However, in the present study, we maintained the same caloric intake for volunteers during monitoring in the 3 study periods. Another plausible mechanism for the reduction in proinflammatory cytokine levels during DIF could be through the insulin-like growth factor 1 (IGF-1), which has been shown to augment proinflammatory cytokine levels [41]. Fasting has been reported to lead to decreased IGF-1 levels in animals [42]. A recent study demonstrated a significant reduction in IGF-1 levels during *Ramadan* DIF compared to baseline [43].

The present study had strengths, as well as limitations. The strength of this study rested in that it was the first study to assess plasma levels and circadian patterns of selected proinflammatory cytokines in volunteers performing DIF during and outside of *Ramadan* while controlling for several confounders that might affect the levels of the measured cytokines. A good control of confounders was achieved during FOR, and this may explain the better results obtained during FOR. Nevertheless, the current study had also inherent limitations that need to be addressed. All experimental studies that have performed several physiological measurements under controlled conditions in a restricted time period (*Ramadan* month), had only a relatively small number of participants included. This has been an inherent limitation of all previous studies that used a similar design and objectively assessed physiological parameters under controlled conditions [9, 19, 44–47]. Another point that needs to be addressed is the fact that this study was conducted on healthy, non-obese young males; hence the current results cannot be extrapolated to females, different age groups or obese subjects with comorbidities. Females were not included in this study, because from a religious point of view, they have to break the fast during the menstrual cycle. Lastly, as the study group was enrolled from within a university, the studied sample may not represent the general public because University employees are more likely to have higher education than the general public.

In summary, in young healthy male volunteers on predetermined caloric intake, set proportions of carbohydrate, fat, and protein consumption and similar light exposure and sleep schedules during baseline and DIF exposure, DIF resulted in significantly decreased plasma levels of cytokines (IL-1β, IL-6, and IL-8), particularly IL-1β and IL-6, across 24 h. However, DIF had no effect on the circadian patterns of the measured cytokines as shown by cosinor analysis. The current findings suggest that DIF might aid in overall health promotion by reducing the levels of proinflammatory cytokines.

Future studies should examine the timely persistence of this reduction in basal cytokines expression levels after *Ramadan* and whether DIF has similar anti-inflammatory effects under conditions of chronic inflammation, such as obesity and other chronic inflammatory disorders.
